# Neurofibromatosis Type 1 with Plexiform Neurofibroma: A Novel Mutation Uncovered

**DOI:** 10.22336/rjo.2026.18

**Published:** 2026

**Authors:** Pragati Garg, Shourriya Garg, Aparajita Shukla, Ruchi Shukla, Shrinkhal

**Affiliations:** 1 Department of Ophthalmology, AIIMS Raebareli, Uttar Pradesh, India; 2 Jhalawar Medical College, Rajasthan, India

**Keywords:** neurofibromatosis, plexiform neurofibroma, genetic variant, whole exome sequencing, Lisch nodules, NF1 = Neurofibromatosis Type 1, WES = Whole Exome Sequencing, LOC = Loss of Consciousness, MPNST = Malignant Peripheral Nerve Sheath Tumor, NGS = Next-Generation Sequencing

## Abstract

**Objectives:**

The objective of this article is to present a classic case of Neurofibromatosis Type 1 (NF1), a multisystemic genetic disorder, in a 16-year-old female who demonstrated hallmark clinical features, including café-au-lait macules, Lisch nodules, and a plexiform neurofibroma. This case also highlights the diagnostic significance of next-generation sequencing (NGS), which identified a novel pathogenic missense variant in the *NF1* gene (c.988G>C; p.Ala330Pro).

**Case presentation:**

A 16-year-old female presented with a 5-year history of progressive, painless right upper eyelid swelling and ptosis. Examination revealed more than six café-au-lait macules, palmar freckling, and Lisch nodules in the right eye. A soft, ill-defined mass involving the right upper eyelid was consistent with plexiform neurofibroma. She also experienced multiple transient episodes of loss of consciousness (LOC), though no neurological deficits were observed. Whole exome sequencing (WES) revealed a novel heterozygous missense variant (c.988G>C; p.Ala330Pro) in exon 9 of the *NF1* gene, classified as likely pathogenic. The variant was absent from population databases and supported by high REVEL and CADD scores. Surgical excision and ptosis correction were performed, and genetic counselling was initiated for the family.

**Discussion:**

NF1 is a common autosomal dominant disorder with variable phenotypic expression and a wide range of complications. Plexiform neurofibromas are among the more severe manifestations and carry a risk of malignant transformation. The novel *NF1* variant identified in this case affects a highly conserved residue and expands the spectrum of NF1 mutations. The clinical and molecular findings underscore the importance of early genetic testing to guide diagnosis, management, and familial risk assessment. The episodes of LOC raise the possibility of neurovascular involvement, which, while rare, is a recognized complication of NF1.

**Conclusion:**

This case underscored the diagnostic value of combining classical clinical features with molecular analysis in NF1. Early detection of pathogenic mutations, appropriate surgical intervention, and long-term follow-up are essential to reduce complications. Multidisciplinary care and emerging targeted therapies, such as MEK inhibitors, may further improve management and prognosis for patients with complex NF1 manifestations.

## Introduction

One of the most common hereditary diseases is neurofibromatosis type 1 (NF1), which is thought to affect 1 in 3,000 people worldwide. NF1 was initially identified by Friedrich von Recklinghausen in 1882. It is caused by mutations in the NF1 gene, located on chromosome 17q11.2 and encoding the tumour suppressor protein neurofibromin. When neurofibromin function is compromised, the RAS/MAPK pathway becomes dysregulated, leading to unchecked cell proliferation [1].

Clinically, NF1 can present with a wide range of symptoms, from mild cutaneous features such as café-au-lait macules to serious consequences, including malignant peripheral nerve sheath tumours (MPNST), plexiform neurofibromas, and optic pathway gliomas [2]. De novo mutations account for about half of cases, with autosomal dominant inheritance accounting for the remainder [3]. Developments in molecular diagnostics, especially next-generation sequencing, have greatly improved our capacity to identify harmful NF1 mutations and to understand their clinical spectrum [4,5].

This case report focused on a patient with classical features of NF1 and a novel pathogenic variant, highlighting the clinical presentation, genetic findings, and management strategies, and contextualizing these within the broader literature.

## Case presentation

**Fig. 1:**
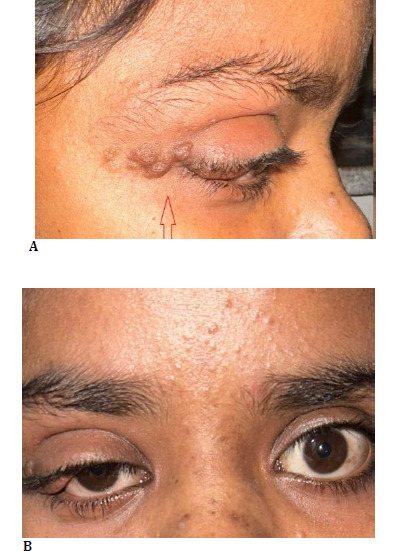
**A, B** Lateral and frontal views showing plexiform neurofibroma (red arrow) involving the right upper eyelid with associated mechanical ptosis

**Fig. 2:**
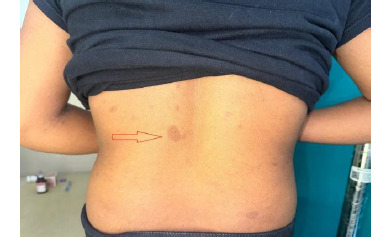
Multiple café-au-lait macules (red arrow) observed on the trunk, characteristic of Neurofibromatosis Type 1

A 16-year-old female presented with a 5-year history of painless, progressive right upper eyelid swelling and ptosis (**Fig. 1**). On examination she exhibited café-au-lait spots (>6) (**Fig. 2**), palmar freckling, and Lisch nodules in the right eye and Plexiform neurofibroma (**Fig. 1A**) causing ptosis of the right upper eyelid (**Fig. 1B**). Her clinical course included 3–4 episodes of transient loss of consciousness (LOC), though neurological deficits were not prominent.

The patient’s family history was significant for similar features in her mother, maternal aunt, and maternal grandfather, suggesting autosomal dominant inheritance.

Whole Exome Sequencing (WES) identified a heterozygous missense variant in the NF1 gene (c.988G>C; p.Ala330Pro) located in exon 9. Computational evidence supported the pathogenicity of the variant (REVEL score: 0.719; CADD score: 27.4). The variant was previously unreported in large population databases (gnomAD) and was classified as likely pathogenic based on ACMG guidelines. Literature review identified a similar variant at the same residue (p.Ala330Val) associated with NF1, underscoring the clinical significance of this amino acid site [6].

**Fig. 3:**
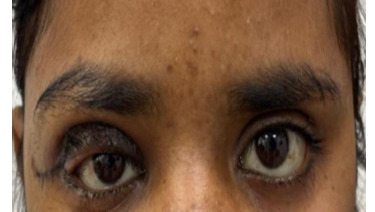
Postoperative view of the right upper eyelid following surgical excision of the neurofibroma and ptosis correction

The clinical and genetic findings confirmed the diagnosis of NF1. Based on the patient’s phenotype and family history, genetic counselling was recommended for the family. Patient underwent tumor excision with management of ptosis (**Fig. 3**) with recommendations for regular follow-up to monitor for complications such as malignant transformation of neurofibromas or other systemic manifestations of NF1 [7].

## Discussion

Neurofibromatosis type 1 (NF1) is a multisystem disorder caused by mutations in the *NF1* gene on chromosome 17, which encodes the tumor suppressor protein neurofibromin. This protein plays a regulatory role in the RAS/MAPK signaling pathway, and loss-of-function mutations disrupt this pathway, leading to uncontrolled cell proliferation and diverse clinical manifestations of NF1 [1].

In the present case, the patient demonstrated classical stigmata of NF1, including café-au-lait spots, Lisch nodules, and a plexiform neurofibroma, fulfilling the NIH diagnostic criteria [2]. A novel heterozygous missense mutation (c.988G>C; p.Ala330Pro) in exon 9 of the *NF1* gene was detected through whole exome sequencing (WES). This variant was classified as likely pathogenic based on multiple criteria, including its conservation across species, computational scores (REVEL: 0.719; CADD: 27.4), and absence from population databases. Moreover, a different missense mutation affecting the same amino acid (p.Ala330Val) has previously been reported in association with NF1, underscoring the importance of this residue [3,4].

Plexiform neurofibromas, as observed in this patient, are among the more severe manifestations of NF1. They can involve critical anatomical structures, potentially leading to pain, disfigurement, and neurological deficits. Although generally benign, these lesions carry a 10% risk of transforming into malignant peripheral nerve sheath tumors (MPNST) [5]. Therefore, careful surveillance with regular imaging and clinical evaluation is warranted, especially in the presence of new or rapidly progressive symptoms [6].

The patient’s episodes of transient loss of consciousness (LOC) were an uncommon feature of NF1 but might suggest neurovascular involvement. Cerebral vasculopathy, although rare, is a recognized manifestation of NF1 and could present with similar symptoms. Hence, neurological imaging and evaluation are essential to rule out central nervous system involvement [7].

The *NF1* gene is one of the most mutation-prone loci in the human genome, with over 3,000 unique variants described to date. These include missense, nonsense, frameshift, splicing mutations, and large deletions [8]. The p.Ala330Pro variant reported in this case contributes to the growing list of pathogenic missense mutations in NF1 [9]. Nemethova et al. highlighted that mutations affecting conserved residues, such as Ala330, are often associated with clinically significant phenotypes, further supporting the relevance of our findings [10].

The diagnostic sensitivity of *NF1* mutation detection has significantly improved with the advent of combined RNA and DNA-based techniques. Evans et al. demonstrated that these methods could detect over 95% of mutations in patients meeting NIH criteria, including those with mosaicism or atypical presentations [4]. In such contexts, next-generation sequencing platforms like WES are invaluable for establishing a definitive diagnosis [11].

NF1 exhibits remarkable phenotypic variability. Café-au-lait macules and Lisch nodules are observed in nearly all affected individuals, while plexiform neurofibromas occur in 30–50% of cases. Other clinical features include cutaneous neurofibromas, optic pathway gliomas, skeletal dysplasias, and learning disabilities. In rare cases, cardiovascular, endocrine, and gastrointestinal involvement may occur [12,13]. Friedman reported that approximately half of all NF1 cases arise from de novo mutations, with the remainder following an autosomal-dominant inheritance pattern [4].

Management of NF1 requires a multidisciplinary approach. While many manifestations are benign and require only observation, symptomatic lesions such as plexiform neurofibromas often necessitate surgical intervention. Recent advances in targeted therapies, particularly MEK inhibitors such as selumetinib, have demonstrated efficacy in reducing the size of inoperable plexiform neurofibromas in pediatric populations, marking a promising shift toward molecularly targeted treatment [14].

Advances in genomic technologies, such as WES, have significantly enhanced understanding and clinical management of NF1. As shown in this case, correlating genotype with phenotype enables better prognostication and personalized care. Furthermore, early identification of pathogenic mutations aids in family counseling, risk stratification, and timely surveillance for complications such as MPNST [7].

Long-term prospective studies are still needed to refine genotype-phenotype correlations and understand the natural history of NF1. Nonetheless, early diagnosis and intervention, bolstered by genetic testing and multidisciplinary follow-up, remain cornerstones for improving outcomes and quality of life in patients with NF1.

## Conclusion

This case highlighted the classic presentation of Neurofibromatosis Type 1 with a plexiform neurofibroma and identified a novel, likely pathogenic NF1 gene variant. The findings underscored the importance of comprehensive clinical evaluation and genetic testing in confirming diagnosis and guiding family counseling. Early detection and multidisciplinary management remain pivotal in reducing morbidity and anticipating complications such as malignant transformation. Novel therapies targeting specific pathways may hold promise for managing complex manifestations of NF1.
